# Quaternized chitosan nanoparticles loaded with the combined attenuated live vaccine against Newcastle disease and infectious bronchitis elicit immune response in chicken after intranasal administration

**DOI:** 10.1080/10717544.2017.1388450

**Published:** 2017-10-13

**Authors:** Kai Zhao, Shanshan Li, Wei Li, Lu Yu, Xutong Duan, Jinyu Han, Xiaohua Wang, Zheng Jin

**Affiliations:** a Key Laboratory of Microbiology, School of Life Science, Heilongjiang University, Harbin, People’s Republic of China;; b School of Biological Science and Technology, University of Jinan, Jinan, People’s Republic of China;; c Department of Avian Infectious Disease, Shanghai Veterinary Research Institute, Chinese Academy of Agricultural Sciences, Shanghai, People’s Republic of China;; d Key Laboratory of Chemical Engineering Process and Technology for High-efficiency Conversion, College of Chemistry and Material Sciences, Heilongjiang University, Harbin, People’s Republic of China

**Keywords:** Newcastle disease, infectious bronchitis, N-2-hydroxypropyl trimethyl ammonium chloride chitosan nanoparticles, vaccine adjuvant, intranasal delivery

## Abstract

Newcastle disease (ND) and infectious bronchitis (IB) are important diseases, which cause respiratory diseases in chickens, resulting in severely economic losses in the poultry industry. In this study, *N*-2-hydroxypropyl trimethyl ammonium chloride chitosan (N-2-HACC) and *N,O*-carboxymethyl chitosan (CMC) were synthesized as adjuvant and delivery carrier for vaccine antigens. N-2-HACC-CMC/NDV/IBV nanoparticles (NPs) (NDV/La Sota and IBV/H120 encapsulated in N-2-HACC-CMC NPs) and N-2-HACC-CMC/NDV-IBV NPs (the mixing of N-2-HACC-CMC/NDV NPs and N-2-HACC-CMC/IBV NPs in a ratio of 1:1) were prepared by the polyelectrolyte composite method, respectively. Both nanoparticles exhibited lower cytotoxicity and higher stability. Their bioactivities were maintained when they were stored at 37 °C for three weeks. Release assay *in vitro* showed that both NDV and IBV could be sustainably released from the nanoparticles after an initial burst release. *In vivo* immunization of chickens showed that N-2-HACC-CMC/NDV/IBV NPs or N-2-HACC-CMC/NDV-IBV NPs intranasally induced higher titers of IgG and IgA antibodies, significantly promoted proliferation of lymphocytes and induced higher levels of interleukine-2 (IL-2), IL-4 and interferon-γ (IFN-γ) than the commercially combined attenuated live vaccine did. This is the first study in the field of animal vaccines demonstrating that intranasal administration of chickens with antigens (NDV and IBV) encapsulated with chitosan derivative could induce humoral, cellular, and mucosal immune responses, which protected chickens from the infection of highly virulent NDV and IBV. This study indicated that N-2-HACC-CMC could be used as an efficient adjuvant and delivery carrier for further development of mucosal vaccines and drugs and could have an immense application potential in medicine.

## Introduction

Newcastle disease (ND) caused by Newcastle disease virus (NDV) is a highly contagious viral disease of poultry and characterized by nervous, respiratory, enteric, and reproductive infections (Kapczynski et al., 2013; Talebi et al., 2015). Similarly, infectious bronchitis (IB) caused by infectious bronchitis virus (IBV) is an acute, highly contagious disease of poultry. Its pathogenesis is characterized by respiratory symptoms, which can usually cause complex infection and secondary bacterial disease, a significant cause of concern to the chicken industry (Awad et al., 2015; Kouakou et al., 2015; Talebi et al., 2015). Incidents of ND and IB infection are relatively high, and these diseases are also more complex, making it harder to prevent and control them. The combined vaccines are therefore used to prevent and control various diseases of chickens.

Currently, most of the combined vaccines are either inactivated or attenuated live vaccines. However, these vaccines have several disadvantages, including poor immunogenicity, reservation of partial virus toxicity, and induction of pathological changes in respiration (Sridhar et al., 2015). DNA vaccines have a number of the advantages, including safety, convenient storage, and transportation. DNA vaccines can stimulate both humoral and cellular immunity etc. (Flingai et al., 2013), however, due to their low expression level and the easy degradation of the plasmid DNA in the body, the antibody levels induced by DNA vaccines are not sufficiently high and the long-term expression protein levels are low. Moreover, they also easily cause immune tolerance. All of these shortages limit the wider application of DNA vaccines, especially in large animal models (Fowler et al., 2012). Therefore, effective strategies are selected to improve the efficacy of vaccines, such as the use of a powerful adjuvant to enhance immunogenicity, optimization of the delivery methods, selection of appropriate routes of immunization, and targeting for effective antigen presentation (Manoj & Babiuk, 2004; Xu et al., 2011; Di Giacomo et al., 2015; Liu et al., 2016).

Use of polymers in vaccine formulations can improve the delivery of antigens and thus can reduce the booster doses of vaccines required for the activation of an appropriate immune response. For many decades, various natural and synthetic biodegradable polymers have been used for antigen delivery for the controlled release of vaccines and as an adjuvant for enhancing immunogenicity of weak vaccines. Additionally, vaccine antigens encapsulated in the nanoparticles administrated *via* the mucosal route can not only protect antigen from being degraded both *in vitro* and *in vivo*, but also ensure the release of the encapsulated antigen at the site of action so as to induce effective immune responses (Sharma et al., 2015).

Chitosan and its derivatives have many advantages, such as low toxicity, environmental compatibility, positive charge, biocompatibility, biodegradability, and sustained release etc. (Huang et al., 2015), which show a great potential for carrying drugs through oral, intranasal and intramuscular administrations. More recently, chitosan and its derivatives have drawn increasing attention because they can serve as an adjuvant and delivery carrier for vaccines or drugs (Rauw et al., 2010). However, when chitosan is subject to a pH≥ 6.5, the number of its positive charges is reduced, thereby decreasing its water solubility. This ultimately affects its delivery efficiency (Wu et al., 2016). Quaternized chitosan has better the water solubility than chitosan does. It retains the original properties of chitosan, including good bioavailability and biocompatibility, low cost, and an ability to open intracellular tight junctions. For instance, Sonia & Sharma (2011) used *N*-2-hydroxypropyl trimethyl ammonium chloride chitosan (N-2-HACC) as a carrier for the delivery of insulin. The results showed that the N-2-HACC nanoparticles had the characteristics of biological adhesion and were able to mediate the slow release of insulin. In addition, confocal microscopic observation also showed that the N-2-HACC nanoparticles could open the tight junctions between cells, indicating that the N-2-HACC can be used as a delivery carrier (Cheng et al., 2013). In addition, *N,O*-carboxymethyl chitosan (CMC) has excellent solubility in water. However, because CMC carries negative charge, it is not conducive to the combination of vaccine antigens; thus, the drugs encapsulated in microspheres or nanoparticles are prepared *via* cross-linking using both CMC and amino group carriers.

In order to enable chitosan to be used more widely, our group has synthesized three water-soluble chitosan derivatives, *O*-2'-hydroxypropyl trimethyl ammonium chloride chitosan (O-2′-HACC), N-2-HACC, and CMC (Jin et al., 2013), and prepared NDV encapsulated in O-2′-HACC nanoparticles by the ionic cross-linking method (Dai et al., 2015). We found that preparation of N-2-HACC was easier and more cost effective than that of O-2′-HACC. Furthermore, we have optimized the synthesis of N-2-HACC, and the N-2-HACC had higher water solubility and more suitable size than chitosan and O-2′-HACC. Currently, there have been few reports on veterinary vaccine antigens encapsulated in chitosan derivatives nanoparticles as an adjuvant and delivery carrier. Herein, we evaluated the optimized biodegradable polymer N-2-HACC as an adjuvant and delivery carrier for veterinary vaccines. In the present study, we prepared the N-2-HACC/CMC/NDV/IBV NPs (NDV/La Sota and IBV/H120 encapsulated in N-2-HACC-CMC nanoparticles) and N-2-HACC-CMC/NDV-IBV NPs (the mixing of N-2-HACC-CMC/NDV NPs and N-2-HACC-CMC/IBV NPs in a ratio of 1:1) by using the polyelectrolyte composite method, respectively. Moreover, we also assessed the characteristics as a delivery carrier for vaccine antigens, and its abilities to induce immune responses and to protect specific pathogen free (SPF) chickens from being infected by NDV or IBV after intranasal administration. This study has paved a good foundation for the further development of mucosal vaccines by using the N-2-HACCand CMC as adjuvant and delivery carrier. The biomaterial-based nanoparticles have promising application potential in the development of new vaccines and drugs in future.

## Materials and methods

### Synthesis of N-2-hydroxypropyl trimethyl ammonium chloride chitosan and N,O-carboxymethyl chitosan

The water soluble chitosan derivatives, *N*-2-hydroxypropyltrimethyl ammonium chloride chitosan (N-2-HACC) and *N,O*-carboxymethyl chitosan (CMC), have been synthesized as the nano-materials for the delivery of vaccine antigen by our laboratory and the chemical structure and characterization of N-2-HACC and CMC have been documented (Jin et al., 2013). Syntheses of both N-2-HACC and CMC were carried out according to the methods described previously (Jin et al., 2013).

### Preparation of N-2-HACC-CMC/NDV NPs and N-2-HACC-CMC/IBV NPs

NDV La Sota strain was provided by Harbin Pharmaceutical Group Bio-Vaccine Co., Ltd. (Harbin, Heilongjiang, China). Encapsulation of NDV into the N-2-HACC-CMC nanoparticles (N-2-HACC-CMC/NDV NPs) was conducted according to the procedures established previously by our group (Jin et al., 2013).

N-2-HACC-CMC/IBV NPs were prepared as follows: IBV was provided by the Harbin Veterinary Research Institute, Chinese Academy of Agricultural Sciences (Harbin, Heilongjiang, China). Encapsulation of IBV into the N-2-HACC-CMC nanoparticles (N-2-HACC-CMC/IBV NPs) was performed with a polyelectrolyte complex method. Briefly, under magnetic stirring at 300 rpm for 3 min, 200 μL of IBV solution was added to 5 mL of N-2-HACC solution (1.0 mg/mL), and then mixed well by magnetically stirring at 1200 rpm for 1 min. Later, 2 mL of CMC solution (0.8 mg/mL) was added drop by drop, and were mixed well by magnetically stirring at 1200 rpm for 40 min. The resulting solution was centrifuged at 12,000 rpm, 4 °C for 20 min. The supernatant was discarded. The nanoparticles were washed three times with 4 °C precooled sterile deionized water. Finally, the resulting nanoparticles were freezing dried for 24 h using a vacuum freeze-drying machine (Boc Edwards Co., Ltd., London, UK). These dried nanoparticles were stored at 4 °C. These nanoparticles obtained were named N-2-HACC-CMC/IBV-NPs.

### Characterization of the N-2-HACC-CMC/NDV NPs and N-2-HACC-CMC/IBV NPs

The morphological and surface characteristics of the N-2-HACC-CMC/NDV NPs and N-2-HACC-CMC/IBV NPs were visualized under JEM-200EX transmission electron microscopy (TEM) (Hitachi Ltd., Tokyo, Japan). The particle size and distribution and zeta potentials were evaluated using a Zeta Sizer 2000 (Malvern Instruments Ltd., Southborough, MA). Samples were diluted with deionized water and the measurements were performed at a scattering angle of 90 degrees and a temperature of 25 °C. The diameters of nanoparticles were calculated from the autocorrelation function of the intensity of light scattered from the particles by assuming that the particles were spherical:

(1) $$\text{EE }\left(\%\right)=\left(W_{0}-W_{1}\right)/W_{0}*100\%,\text{ LC }\left(\%\right)=\left(W_{0}-W_{1}\right)/W_{N}\times 100\%$$

where *W*_0_ is total amount of NDV or IBV added, *W*_1_ is amount of the free NDV or IBV, and *W*_N_ is the weight of nanoparticles. All the measurements were performed five times.

### Viral titers in the N-2-HACC-CMC/NDV NPs, N-2-HACC-CMC/IBV NPs, and commercially combined attenuated live vaccine against Newcastle disease and infectious bronchitis

Viral titers were calculated by measuring the 50% embryo infectious dose (EID_50_) as previously described (Barakat & Almurshedi, 2011). EID_50_ was calculated according to Reed–Muench method (Olabode & Ndako, 2010). Briefly, 100 μg of the N-2-HACC-CMC/NDV NPs or N-2-HACC-CMC/IBV NPs were added into 10 mL of PBS buffer (pH 7.2). Subsequently, 2.0 mL of trypsin (Sigma-Aldrich, Inc, St. Louis, MO) was added to the above solution, which was then digested for 72 h at 4 °C with a final concentration of trypsin solution at 0.25% and centrifuged at 1200 rpm for 5 min. The obtained supernatant was diluted in a 10-fold series with sterilized saline. The 10^−5^, 10^−6^, 10^−7^, and 10^−8^ dilutions (the dilutions for IBV titers were 10^−4^, 10^−5^, 10^−6^, and 10^−7^) were inoculated into the allantoic cavity of five 10-day-old specific pathogen free (SPF) chicken embryonated eggs from the Harbin Veterinary Research Institute, Chinese Academy of Agricultural Sciences (Harbin, Heilongjiang, China), respectively, and incubated at 37 °C for 120 h. Dead embryos at 24 h post-inoculation were discarded. Eggs were chilled at 4 °C. The allantoic fluid from the remaining eggs was harvested and tested for hemagglutination (HA). Harvest of allantoic fluid performed as follows: before harvesting the allantoic fluid, the eggs were chilled at 4 °C for at least 2 h to kill the embryo and to reduce the contamination of the allantoic fluid with blood during harvest. Each egg was swabbed with cotton wool presoaked with 70% alcohol to disinfect and remove condensation from the shells. Later, the eggshell above the air space was removed by using the dip of the forceps or scissors which were presoaked in 100% alcohol and flamed to sterilize, and embryos that were visibly contaminated were discarded; A sample of allantoic fluid was removed from each egg and each sample was tested for the presence of the virus by the hemagglutination test, and embryos that were not positive to HA for the virus were discarded. Finally, the allantoic fluid was harvested from the eggs and transferred into sterile containers by using sterile glass Pasteur pipettes. All the dilutions of virus or antisera were made in PBS. HA titers were expressed as log2 of the reciprocal of the highest dilution at which the HA pattern covered at least 75% of the area of full hemagglutination. The results were regarded as positive when HA titers were equal to or greater than 7.0 log2.

Commercially combined attenuated live vaccine against ND and IB (La Sota + H120) was provided by Harbin Pharmaceutical Group Bio-vaccine Co., Ltd (Harbin, Heilongjiang, China). The combined attenuated live vaccine was dissolved in PBS buffer (pH 7.4), and the solution was neutralized with IBV positive serum obtained from Harbin Veterinary Research Institute, China Academy of Agricultural Sciences (Harbin, Heilongjiang, China). Later, by a serial of 10-fold dilution with PBS buffer, the 10^−6^, 10^−7^, 10^−8^, and 10^−9^ dilutions were taken to detect NDV content. Meanwhile, the dissolution of combined attenuated live vaccine was neutralized with NDV positive serum obtained from the Harbin Veterinary Research Institute, China Academy of Agricultural Sciences. After a serial of 10-fold dilution, the 10^−4^, 10^−5^, 10^−6^, and 10^−7^ dilutions were taken to detect the IBV content.

### *In vitro* release of the N-2-HACC-CMC/NDV NPs,N-2-HACC-CMC/IBV NPs

The assays of the release of NDV and IBV from the nanoparticles were carried out by the Bicinchoninic Acid Protein Assay Kit as previously described (Zhao et al., 2014). Briefly, 0.1 g of freezing-dried N-2-HACC-CMC/NDV NPs or N-2-HACC-CMC/IBV NPs were added to 2 mL of PBS buffer (pH 7.4), then put into the constant temperature shaking table and fully stirred at 1000 rpm, 37 °C. Sample solution (1 mL) was withdrawn at predetermined time intervals (0, 4, 8, 12, 16, 20, 24, 36, 48, 60, 72, 84, 96, 108, 120, 132, 144, 156 and 168 h) and centrifuged at 13,000 rpm and 4 °C for 30 min. The absorbance at 595 nm (OD_595_) was measured by spectrophotometer. The release profile was plotted using release time as the *X*-axis and cumulative release amount as the *Y*-axis.

### Preparation of N-2-HACC-CMC/NDV-IBV NPs andN-2-HACC-CMC/NDV/IBV NPs

According to the amounts of NDV and IBV in the N-2-HACC-CMC/NDV NPs, N-2-HACC-CMC/IBV NPs and commercially combined attenuated live vaccine, N-2-HACC-CMC/NDV-IBV NPs were prepared by mixing the N-2-HACC-CMC/NDV NPs and N-2-HACC-CMC/IBV NPs suspension at the ratio of 1:1. After mixing NDV and IBV at the ratio of 1:1, N-2-HACC-CMC/NDV/IBV NPs were obtained according to the preparation of N-2-HACC-CMC/NDV NPs (Jin et al., 2013). The amounts of NDV and IBV in the N-2-HACC-CMC/NDV-IBV NPs or N-2-HACC-CMC/NDV/IBV NPs were the same as those of the commercially combined attenuated live vaccine.

### Safety of the N-2-HACC-CMC/NDV-IBV NPs andN-2-HACC-CMC/NDV/IBV NPs

#### *In vitro* cytotoxicity

The cytotoxicity of N-2-HACC-CMC/NDV-IBV NPs and N-2-HACC-CMC/NDV/IBV NPs was evaluated by chicken embryo fibroblast (CEF) obtained from the Harbin Veterinary Research Institute using the Cell Counting Kit-8 (CCK-8) reagent (Dojindo Ltd, Kumamoto, Japan). CEF cells were cultured in Dulbecco's Modified Eagle's Medium (DMEM) containing 10% fetal bovine serum (FBS) and 1% streptomycin (Gibco BRL, Grand Island, NY) and then diluted to 2 × 10^6^/mL. Cells were transferred to 96-well plates at 200 µL each well and cultured at 37 °C for 5 h. About 100 µL (10^5.1^ EID_50_/0.1 mL) of N-2-HACC-CMC/NDV-IBV NPs or N-2-HACC-CMC/NDV/IBV NPs (diluted in DMEM culture at 1 mg/ml) were added into the wells, followed by incubation at 37 °C for 24 h. Wells containing DMEM alone or CCK-8 and DMEM were used as the controls. About 10 µL of WST-8 reagent (Dojindo Ltd, Kumamoto, Japan) was added into each well, and then incubated 37 °C for 4 h. OD_450_ was measured, and the survival rate of cells was calculated as follows:

(2) Survival rate (%)=[(As-Ab)]/(Ac-Ab)]×100%

where As represents the OD_450_ of test wells containing cell medium, WST-8 and N-2-HACC-CMC/NDV-IBV NPs or N-2-HACC-CMC/NDV/IBV NPs, respectively; Ab represents the OD_450_ of blank wells containing cell medium alone, and Ac represents the OD_450_ of control wells containing cell medium and WST-8.

#### Safety assay

Thirty 14-day-old SPF chickens obtained from Animal Center of Harbin Veterinary Research Institute Laboratory were randomly divided into three groups and chickens in each group were separately housed in a stainless steel isolator with a temperature- and light-controlled environment and free access to food and water ad *libitum*. Chickens in Group 1 were immunized intranasally (i.n.) with 1.0 mL of N-2-HACC-CMC/NDV/IBV NPs; Chickens in Group 2 were immunized i.n. with 1.0 mL of N-2-HACC-CMC/NDV-IBV NPs; Chickens in Group 3 were immunized i.n. with 1.0 mL of PBS. The immunized chickens were continuously observed daily for 14 days, and any abnormal changes including feeding, drinking, mental state and body weight were recorded.

### Storage stability of the N-2-HACC-CMC/NDV-IBV NPs and N-2-HACC-CMC/NDV/IBV NPs

To evaluate the stability of N-2-HACC-CMC/NDV-IBV NPs and N-2-HACC-CMC/NDV/IBV NPs during the storage, these nanoparticles were placed at room temperature, 4, −20, and 37 °C for 21 days, respectively. Their morphological changes were observed and recorded.

### Nasal immunization studies

A total of two hundreds of 4-week-old SPF chickens obtained from Animal Center of Harbin Veterinary Research Institute Laboratory were randomly assigned to five groups (*n* = 40 chickens/group) and group chickens in each group were separately housed in a stainless steel isolator in a temperature- and light-controlled environment with free access to food and water *ad libitum*. Animals were sacrificed by an overdose of a mixture of isoflurane/O_2_. The experimental protocols were approved by the Animal Ethics Committee as stipulated in the guide to the care and use of experimental animals of the Harbin Veterinary Research Institute of the Chinese Academy of Agricultural Sciences. The chickens were euthanized by intravenous injection of pentobarbital.

Chickens in Group 1 were treated with PBS buffer i.n.; Chickens in Group 2 were treated with blank N-2-HACC-CMC NPs i.n. Chickens in Group 3 were treated with the commercially combined attenuated live vaccine against ND and IBD i.n. Chickens in Groups 4 and 5 were intranasally administrated with N-2-HACC-CMC/NDV-IBV NPs and N-2-HACC-CMC/NDV/IBV NPs, respectively, and used to evaluate immune responses against NDV and IBV. Each chicken received 100 μL doses *via* intranasal route.

#### Sera and tissue collection

Blood samples were collected from the wing veins of five chickens in each of the five groups at 1, 2, 3, 4, 5, 6, 7, 8, 9, and 10 weeks post immunization. The sera samples were separated by centrifugation at 3000 rpm at 4 °C for 10 min for immunoglobulin assessment. The intestine, glandular stomach and spleen of these animals were carefully dissected out, washed in sterile PBS and stored at 4 °C for further studies.

#### Assays of hemagglutination inhibition (HI) antibody and IgA antibody

The titers of the specific HI antibody in sera were detected by HI test (*n* = 5). To evaluate the mucosal immune response, serum, tracheal fluid, and Harderian glands were collected from three chickens at 1, 2, 3, 4, 5, 6, 7, 8, 9, and 10 weeks post immunization. Mucosal extracts were obtained by centrifugation at 3000 rpm, 4 °C for 10 min and the supernatant was collected. The titers of IgA antibody were measured by ELISA-sandwich technique according to the instruction manual of NDV and IBV IgA ELISA Kit (Rapidbio Co. Ltd, Beijing, China) (*n* = 3).

#### Assay of lymphocyte proliferation

To assess the cellular-mediated immune responses at 1, 2, 3, 4, 5, 6, 7, 8, 9, and 10 weeks post immunization, proliferation of lymphocytes of the immunized chickens was assayed using MTT colorimetric assay as previously described (Dai et al., 2015). Splenic lymphocytes were prepared from all the experimental chickens using the standard protocol. Briefly, the spleens of these chickens were dissected out and minced in PBS on a stainless steel mesh (−4 μm) to make a singled cell suspension. The erythrocytes were lysed with 0.54% NH_4_Cl (pH 7.4). After being centrifuged, the cells were re-suspended in complete RPMI media supplemented with 10% FBS (Gibco BRL, Grand Island, NY) and diluted to 2 × 10^7^ cells/mL. About 100 μL of the cell suspensions and 100 μL of RPMI media supplemented with 10% FBS were seeded into each well of 96-well culture plates. Wells containing 10 μg/mL ConA (Sigma-Aldrich, Inc, St. Louis, MO) were used as the positive controls; wells without ConA were used as the negative control. All the cells were cultured at 5% CO_2_ and 37 °C for 72 h. Two hour prior to termination, 20 μL of MTT (5 μg/mL) was added into each well. After the appearance of purple formazan crystal, the culture plate was centrifuged. The supernatant was removed and the crystals were dissolved in the 100 μL of dimethyl sulphoxide (DMSO) (Sigma-Aldrich, Inc., St. Louis, MO) and OD_570_ was measured to determine the stimulation indices (SIs), which were determined using the following formula:

(3) $$\text{SIs}={\text{OD}}_{\text{57}0}\text{ T}/{\text{OD}}_{\text{57}0}\text{ C}$$

where OD_570_ T is the mean value of the tested groups, including N-2-HACC-CMC/NDV-IBV NPs, N-2-HACC-CMC/NDV/IBV NPs, the commercial combined attenuated live vaccine, N-2-HACC-CMC NPs; OD_570_ C is the mean value of the PBS group. All the experiments were performed in triplicate and repeated twice with three chickens each.

#### Measurements of secretion levels of interferon-γ (IFN-γ), interleukin-2 (IL-2), and IL-4 in serum

For assays of inflammatory cytokines in serum, serum samples were collected from three chickens at 1, 2, 3, 4, 5, 6, 7, 8, 9, and 10 weeks post immunization. An enzyme-linked immunosorbent assay (ELISA) Kit for chicken IFN-γ, IL-2, and IL-4 (Thermo Fisher Scientific Inc., Waltham, MA) was used to measure the concentrations of IFN-γ, IL-2, and IL-4 in culture supernatants of spleen cells according to the manufacturer’s instructions, respectively. Briefly, culture supernatants from splenocytes derived from both immunized and controlled chickens were diluted in 1:50 with PBS, and 100 μL of the resulting solution was added to triplicate wells of the ELISA plate. The OD_550_ and OD_450_ were measured in a Microplate Reader (Bio-Rad Instruments, Hercules, CA). The former value was then subtracted from the latter. A standard curve was constructed using a set of standards provided by the manufacturer. The experimental values were calculated based on reading of this curve. All the operations were performed according to the procedures described for the cytokine ELISA kits.

#### *In vitro* protective efficacy

The ability of N-2-HACC-CMC/NDV-IBV NPs or N-2-HACC-CMC/NDV/IBV NPs to protect chickens against the infection of NDV strain F48E9 obtained from the Harbin Pharmaceutical Group Bio-vaccine Co. Ltd and IBV strain M41 obtained from the Harbin Veterinary Research Institute was determined. When the levels of NDV-specific antibody and IBV-specific antibody in serum of every immune group reached to 6.0 log2 post immunization, eight chickens were selected randomly from each of the five groups and infected intramuscularly (i.m.) with 100 μL of the highly virulent NDV strain F48E9 (10^4.5^ EID_50_/0.1 mL) and IBV strain M41 (10^4.5^ EID_50_/0.1 mL) and used for challenge studies.

Feed, water drinking, mental state, body weight, clinical symptoms, and mortality of these chickens were continuously observed and recorded for 5 weeks. The infected chickens and chickens in corresponding negative control groups were euthanized and their intestine and glandular stomach were collected and examined by histological staining.

### Statistical analysis

The immunization experiments were repeated three times under the same conditions. Data are presented as mean ± standard deviation (SD). One-way analysis of variance (ANOVA) statistical test with Tukey’s post-hoc test was performed using Origin 7.5 software (OriginLab Corporation, Northampton, MA) to determine the significance of the differences between various groups. *p* Values ≤ .05 were considered as significant.

## Results

### Characterization of the N-2-HACC-CMC/NDV NPs and N-2-HACC-CMC/IBV NPs

Transmission electron microscopy (TEM) showed that the optimal N-2-HACC-CMC/NDV NPs and N-2-HACC-CMC/IBV NPs displayed a spherical morphology (Supplementary Figure S1). The average particle sizes of N-2-HACC-CMC/NDV NPs and N-2-HACC-CMC/IBV NPs were 251.8 ± 10.2 nm and 122.4 ± 4.2 nm (Supplementary Figure S1). Their polydispersity indexes (PDIs) were 0.428 and 0.070 (Supplementary Figure S1) and zeta potentials were 46.6 ± 6.4 mV and 53.2 ± 6.68 mV, respectively (Supplementary Figure S1). The particle size and zeta potentials of N-2-HACC were 103.2 ± 1.6 nm and 38.1 ± 2.7 mV, respectively. Their EEs were 96.10 ± 1.13% and 95.56 ± 0.28% and their LCs were 53.21 ± 0.97% and 52.13 ± 1.52%, respectively (*n* = 3).

### Viral titers in the N-2-HACC-CMC/NDV NPs, N-2-HACC-CMC/IBV NPs and commercially combined attenuated live vaccine

The viral titers in the N-2-HACC-CMC/NDV NPs and N-2-HACC-CMC/IBV NPs were 10^7.4^ EID_50_/0.1 mL and 10^5.5^ EID_50_/0.1 mL, respectively (*n* = 3), which met the manufacturing standards for the attenuated live vaccine as required by the Veterinary Biological Product Rules of the People’s Republic of China (≥10^5^ EID_50_). The amounts of NDV and IBV in the commercially combined attenuated live vaccine were 10^8.5^ EID_50_/0.1 mL and IBV was 10^6.4^ EID_50_/0.1 mL, respectively.

### *In vitro* release of the N-2-HACC-CMC/NDV NPs and N-2-HACC-CMC/IBV NPs from the nanoparticles

NDV and IBV were released from the nanoparticles with burst releases of 56.7 ± 4.3% and 55.6 ± 2.7% within 48 h, respectively. At 96 h, the release rates were 72 ± 4.3% and 70 ± 4.3% of the payload. This was followed by a slow and sustained release until 168 h, and approximately 80.2 ± 5.1% of all the NDV and IBV encapsulated in the both nanoparticles were released.

### Preparation of N-2-HACC-CMC/NDV-IBV NPs and N-2-HACC-CMC/NDV/IBV NPs

In order to obtain the same the amount of antigen as that in the commercially combined attenuated live vaccine, the volumes of the prepared N-2-HACC-CMC/NDV NPs and N-2-HACC-CMC/IBV NPs suspension were reduced by 10 times by centrifugation at 4 °C and 12,000 rpm and 4 °C for 20 min. The N-2-HACC-CMC/NDV-IBV NPs were formed by combining the concentrated N-2-HACC-CMC/NDV-IBV NPs and N-2-HACC-CMC/NDV/IBV NPs in the ratio of 1:1. The concentrations of NDV and IBV in the N-2-HACC-CMC/NDV-IBV NPs were 10^8.4^ EID_50_/0.1 mL and 10^6.5^ EID_50_/0.1 mL, respectively.

NDV and IBV suspensions were mixed in the ratio of 1:1, and then N-2-HACC-CMC/NDV/IBV NPs were prepared according to the preparation method of N-2-HACC-CMC/NDV-IBV NPs. The concentrations of NDV and IBV in N-2-HACC-CMC/NDV/IBV NPs were 10^8.4^ EID_50_/0.1 mL and 10^6.4^ EID_50_/0.1 mL, respectively.

### *In vitro* and *in vivo* cytotoxicity of the N-2-HACC-CMC/NDV-IBV NPs and N-2-HACC-CMC/NDV/IBV NPs

To test safety of the N-2-HACC-CMC NPs as adjuvant and delivery carrier for mucosal immune delivery system, tests of both *in vitro* and *in vivo* cytotoxicity were performed. The survival rates of chicken embryo fibroblast in the N-2-HACC-CMC/NDV-IBV NPs and N-2-HACC-CMC/NDV/IBV NPs were 91.3 ± 2.4% and 90.8 ± 4.1%, respectively, and no significant changes in cell morphology were observed as compared to those of the control cells (*p* > .05).

The *in vivo* cytotoxicity analysis demonstrated that the feeding, drinking, mental state, and body weight of chickens immunized i.n. with the N-2-HACC-CMC/NDV-IBV NPs or N-2-HACC-CMC/NDV/IBV NPs were normal as compared with those of the chickens in control groups. The morbidity and mortality rates of chickens were 0% in the both groups, implying that immunization of the chickens with a high dose of NDV-IBV-N-2-HACC/CMC-NPs or NDV/IBV-N-2-HACC/CMC-NPs is relatively safe. These results showed that the N-2-HACC-CMC NPs as adjuvant and delivery carrier had little cytotoxicity but high safety level when being administrated *via* intranasal route.

### Storage stability of the N-2-HACC-CMC/NDV-IBV NPs and N-2-HACC-CMC/NDV/IBV NPs

N-2-HACC-CMC/NDV-IBV NPs or N-2-HACC-CMC/NDV/IBV NPs were loose, sponge-like and milky white powder. After being stored at the room temperature, 4, −20, and 37 °C for 21 days, no significant changes in the morphology of nanoparticles were observed, indicating that these nanoparticles have good storage stability and can be stored for a long period of time even at the room temperature.

### N-2-HACC-CMC/NDV-IBV NPs and N-2-HACC-CMC/NDV/IBV NPs elicit immune responses in chickens after intranasal administration

#### Serum IgG titers

**Figure 1.**
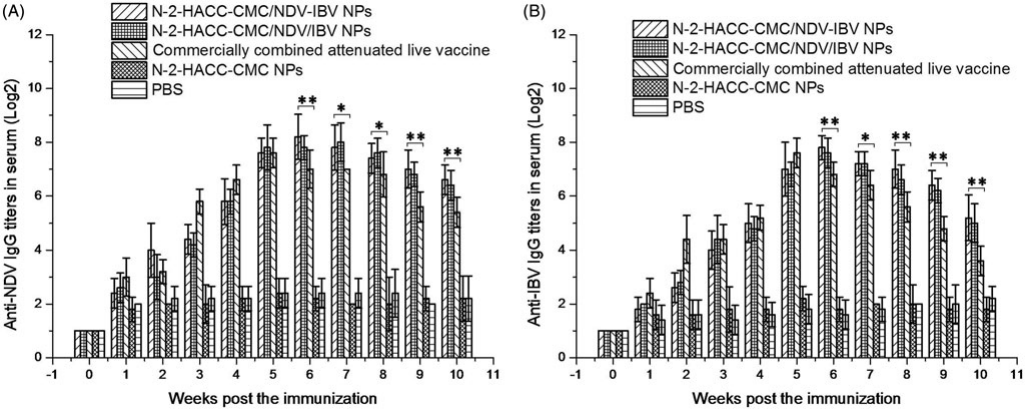
Serum IgG antibody titers following intranasal administration of N-2-HACC-CMC/NDV-IBV NPs, N-2-HACC-CMC/NDV/IBV NPs, commercially combined attenuated live vaccine, N-2-HACC-CMC NPs and PBS. (A) Titers of serum anti-NDV IgG; (B) Titers of serum anti-IBV IgG. Values represent mean ± SD (*n* = 3). *p < .05 and **p < .01 indicate statistically significant differences when compared to commercially combined attenuated live vaccine.

In order to evaluate the immune response of N-2-HACC-CMC nanoparticles after intranasal administration as a novel adjuvant and delivery carrier for vaccines, we detected the immune responses of chickens. As shown in Figure 1, either the N-2-HACC-CMC/NDV-IBV NPs, N-2-HACC-CMC/NDV/IBV NPs or commercially combined attenuated live vaccine induced significant antibody responses in chickens when they were immunized intranasally, and the antibody titers of chickens were quickly increased at the 1–5 weeks post immunization. After the fifth week, chickens immunized with the N-2-HACC-CMC/NDV-IBV NPs or N-2-HACC-CMC/NDV/IBV NPs produced higher titers of anti-NDV IgG and anti-IBV IgG than those immunized with the commercially combined attenuated live vaccine until the 10th week (*p* < .05). It is worth mentioning that titers of anti-NDV and anti-IBV IgG in chickens immunized with N-2-HACC-CMC/NDV/IBV NPs induced higher than those in chickens immunized with N-2-HACC-CMC/NDV-IBV NPs until the 10th week but the difference were not significant (*p* > .05), indicating that both N-2-HACC-CMC/NDV-IBV NPs and N-2-HACC-CMC/NDV/IBV NPs are able to elicit the immune responses of chickens and to maintain higher antibody levels for a long period of time.

##### The immunoglobulin A (sIgA) antibody titers in mucosa extracts

**Figure 2.**
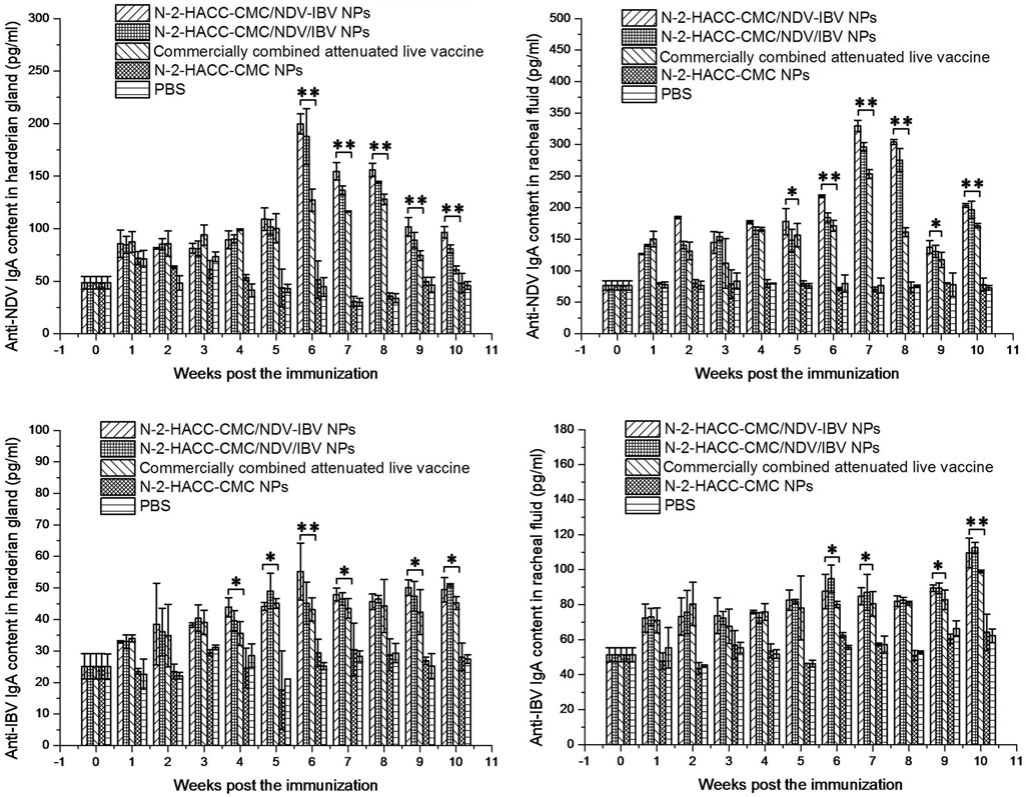
Titers of IgA antibody in tracheal fluid and Harderian gland of SPF chickens nasally immunized with the N-2-HACC-CMC/NDV-IBV NPs, N-2-HACC-CMC/NDV/IBV NPs, commercially combined attenuated live vaccine, N-2-HACC-CMC NPs, and PBS. (A) Content of anti-NDV IgA in Harderian gland; (B) Content of anti-NDV IgA in tracheal fluid; (C) Content of anti-IBV IgA in Harderian gland; (D) Content of anti-IBV IgA in tracheal fluid. The contents of IgA antibody in these samples were detected with ELISA. Values represent mean ± SD (*n* = 3). *p < .05 and **p < .01 indicate statistically significant differences when compared to commercially combined attenuated live vaccine.

The titers of anti-NDV sIgA antibody in chickens immunized with N-2-HACC-CMC/NDV-IBV NPs and N-2-HACC-CMC/NDV/IBV NPs were significantly higher in the Harderian gland (Figure 2(A)) and tracheal fluid (Figure 2(B)), and the secretion periods of anti-NDV sIgA antibody were also longer than those of chickens in the commercially combined attenuated live vaccine group (*p* < .01). Simultaneously, the anti-NDV sIgA antibody levels in the tracheal fluid were significantly higher than in the Harderian gland (*p* < .01).

Among the N-2-HACC-CMC/NDV-IBV NPs, N-2-HACC-CMC/NDV/IBV NPs and commercially combined attenuated live vaccine groups, the lowest anti-IBV sIgA titers were observed in the commercially combined attenuated live vaccine group (Figure 2(C,D)). Both nanoparticles showed significantly higher sIgA titers in the Harderian gland than the commercially combined attenuated live vaccine group did (Figure 2(C), *p* < .05). The titers of anti-NDV sIgA and anti-IBV sIgA in either Harderian gland or tracheal fluid induced with the N-2-HACC-CMC/NDV-IBV NPs and N-2-HACC-CMC/NDV/IBV NPs were not significantly different (*p* > .05).

##### Proliferation of lymphocytes

**Figure 3.**
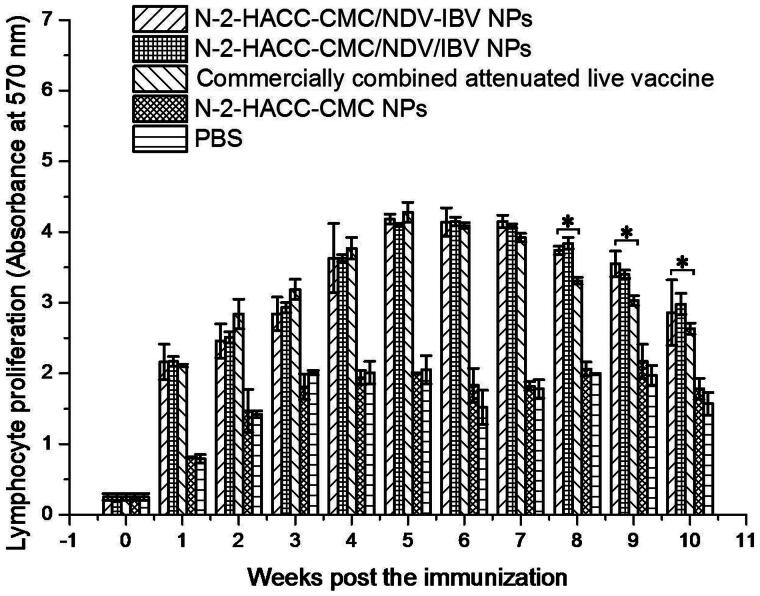
Analysis of proliferation of lymphocytes in the SPF chickens nasally immunized with the N-2-HACC-CMC/NDV-IBV NPs, N-2-HACC-CMC/NDV/IBV NPs, commercially combined attenuated live vaccine, N-2-HACC-CMC NPs, and PBS. Values represent mean ± SD (*n* = 3). *p < .05 and **p < .01 indicate statistically significant differences when compared to commercially combined attenuated live vaccine.

Proliferation of lymphocytes is one of the fundamental characteristics of the response of lymphocytes to antigenic stimulation. Figure 3 revealed that no significant differences in proliferation of lymphocytes were observed among chickens immunized with the N-2-HACC-CMC/NDV-IBV NPs, N-2-HACC-CMC/NDV/IBV NPs and commercially combined attenuated live vaccine groups at 1–7 weeks post immunization (*p* > .05). However, the SIs of chickens immunized with the N-2-HACC-CMC/NDV-IBV NPs and N-2-HACC-CMC/NDV/IBV NPs were significantly higher than those in chickens immunized with the commercially combined attenuated live vaccine after the seventh week (*p* < .05). These results showed that intranasal immunization with the both nanoparticles led to greater and longer proliferation of lymphocytes than the commercially combined attenuated live vaccine did.

##### Induction of cytokines

**Figure 4.**
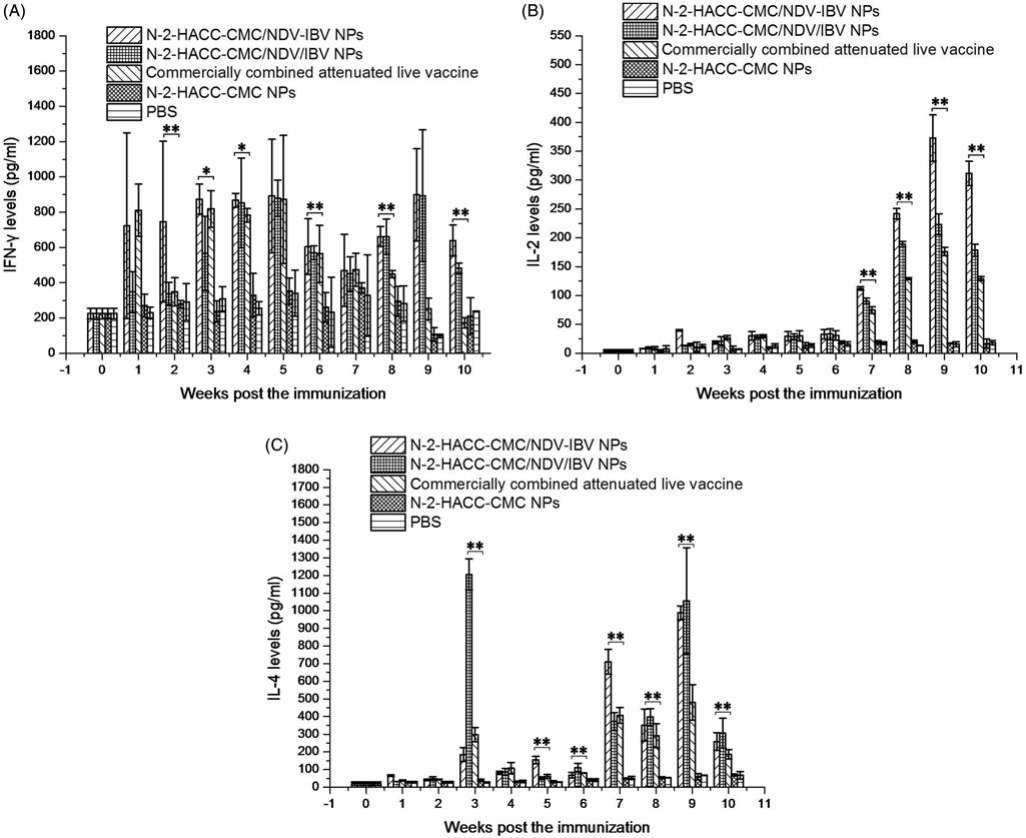
Levels of IFN-γ (A), IL-2 (B), and IL-4 (C) in the supernatant of splenocytes harvested from the SPF chickens nasally immunized with the N-2-HACC-CMC/NDV-IBV NPs, N-2-HACC-CMC/NDV/IBV NPs, commercially combined attenuated live vaccine, N-2-HACC-CMC NPs, and PBS. The levels of IFN-γ, IL-2 and IL-4 in the supernatant were analyzed in an enzyme-linked immunosorbent assay for chicken IFN-γ, IL-2 and IL-4. Values represent mean ± SD (*n* = 3). *p < .05 and **p < .01 indicate statistically significant differences when compared to commercially combined attenuated live vaccine.

An enzyme-linked immunosorbent assay was used to measure the concentrations of inflammatory cytokines, including IFN-γ, IL-2, and IL-4, in supernatants of cultured spleen cells. As shown in Figure 4, the immunization with N-2-HACC-CMC/NDV-IBV NPs and N-2-HACC-CMC/NDV/IBV NPs i.n. triggered significantly higher levels of IFN-γ, IL-2, and IL-4 than those induced by immunization with the commercially combined attenuated live vaccine or the control chickens (*p* < .05), and the levels of IFN-γ in chicken immunized with N-2-HACC-CMC/NDV-IBV NPs were significantly higher than those of chickens in N-2-HACC-CMC/NDV/IBV NPs group at the second, eighth and tenth weeks (Figure 4(A)) and IL-2 (Figure 4(B)) (*p* < .01). However, the levels of IL-4 (Figure 4(C)) in the chickens immunized with N-2-HACC-CMC/NDV-IBV NPs were significantly higher than those in chickens immunized with N-2-HACC-CMC/NDV-IBV NPs (*p* < .05). These results suggested that the both nanoparticles i.n. enhanced the humoral and cellular immune responses than immunization with the commercially combined attenuated live vaccine.

##### Protective effect

The highly virulent NDV strain F_48_E_9_ had killed all the chickens treated with PBS or N-2-HACC-CMC NPs in the 2–6 days after challenge. One chicken in the commercially combined attenuated live vaccine group died at the seventh day, and no dead chickens were found in the both groups administrated with nanoparticles (Supplementary Table S1). Thus, the protective efficacy was 100, 100, and 90% for the chickens immunized with the N-2-HACC-CMC/NDV-IBV NPs, N-2-HACC-CMC/NDV/IBV NPs, and commercially combined attenuated live vaccine, respectively. Feeding, drinking, mental state, and body weight were normal for chickens treated with the both nanoparticles, but were abnormal for two chickens treated with the commercially combined attenuated live vaccine. However, after being challenged with the IBV strain M41, all seven chickens in PBS group were sick and died, six chickens in the N-2-HACC-CMC NPs group were sick and died, and only one chicken in the both commercial vaccine and N-2-HACC-CMC/NDV/IBV NPs showed a light symptom but did not die (Supplementary Table S1). No pathological and histopathological changes were found in the intestine and glandular stomach collected from the chickens immunized with the both nanoparticles groups after being challenged with either strain F_48_E_9_ or strain M41, but the typical pathological and histopathological changes were seen in the dead chickens from the PBS and N-2-HACC-CMC NPs groups (Supplementary Figure S2).

**Figure 5.**
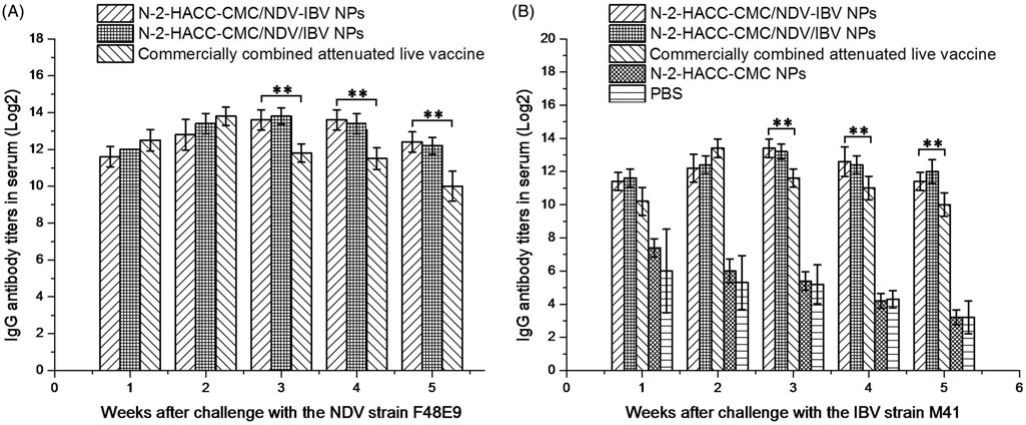
Titers of anti-NDV IgG (A) and titers of anti-IBV IgG (B) in serum of the immunized SPF chickens after being challenged with the highly virulent NDV strain F48E9 and IBV strain M41. Values represent mean ± SD (*n* = 3). *p < .05 and **p < .01 indicate statistically significant differences when compared to commercially combined attenuated live vaccine.

**Figure 6.**
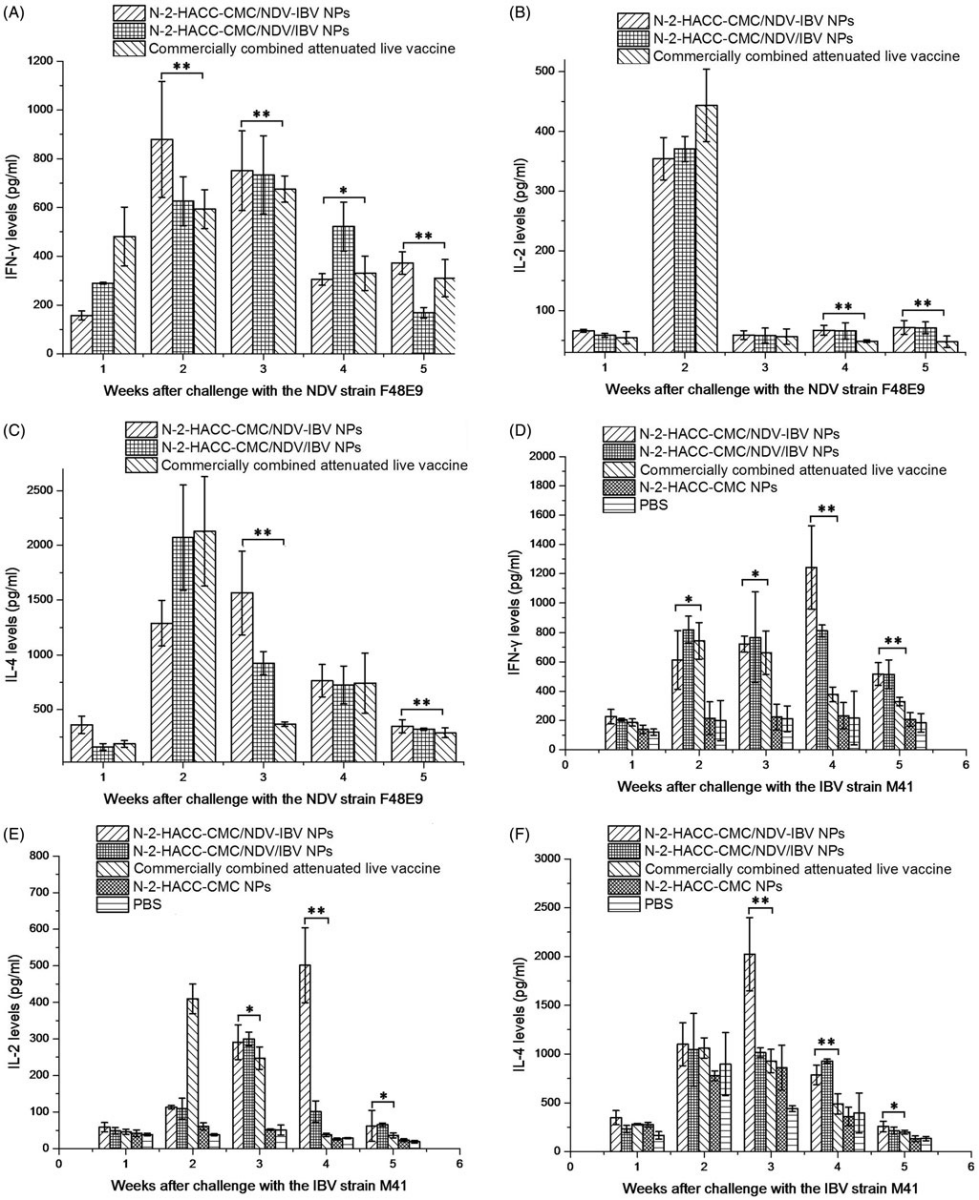
Levels of IFN-γ, IL-2, and IL-4 in the supernatant of splenocytes harvested from the immunized SPF chickens after being challenged with the highly virulent NDV strain F48E9 and IBV strain M41. A, B and C represent the levels of IFN-γ, IL-2, and IL-4 after being challenged with the NDV strain F48E9, respectively; D, E, and F represent levels of IFN-γ, IL-2, and IL-4 after being challenged with the IBV strain M41, respectively. The levels of IFN-γ, IL-2, and IL-4 in the supernatant were analyzed in an enzyme-linked immunosorbent assay for chicken IFN-γ, IL-2, and IL-4. Values represent mean ± SD (*n* = 3). *p < .05 and **p < .01 indicate statistically significant differences when compared to commercially combined attenuated live vaccine.

After challenge with the strain NDV F48E9 and IBV M41, the titers of anti-NDV and anti-IBV IgG antibody in serum of chickens immunized with the N-2-HACC-CMC/NDV-IBV NPs, N-2-HACC-CMC/NDV/IBV NPs and commercially combined attenuated live vaccine were significantly increased, and the both nanoparticles resulted in higher serum IgG titers (*p* < .05) after the third week, and could maintained for a longer period of time and were more slowly released as compared with those of the commercially combined attenuated live vaccine group (Figure 5). Simultaneously, Figure 6(A–F) showed that the levels of IFN-γ, IL-2, and IL-4 were significantly higher in the chickens immunized with the N-2-HACC-CMC/NDV-IBV NPs or N-2-HACC-CMC/NDV/IBV NPs than in chickens immunized with the commercially combined attenuated live vaccine or the control chickens (*p* < .05). But no difference in the IL-2 levels (Figure 6(B)) was detected between the two groups from the third week (*p* > .05). The levels of IFN-γ (Figure 6(A)) and IL-4 (Figure 6(C)) at the fifth week after challenge with the strain NDV F48E9 were significantly different between the N-2-HACC-CMC/NDV-IBV NPs and N-2-HACC-CMC/NDV/IBV NPs (*p* < .05), while the levels of IFN-γ (Figure 6(D)) and IL-2 (Figure 6(E)) at the third and fifth weeks after challenge were not significantly different between the two groups (*p* > .05), the levels of IFN-γ (Figure 6(D)) and IL-2 (Figure 6(E)) at the fourth week were higher in chickens immunized with N-2-HACC-CMC/NDV-IBV NPs than those of chickens immunized with N-2-HACC-CMC/NDV/IBV NPs (*p* < .05).

## Discussion

Nasal immunization is a mucosal administration route, which shows considerable potential for vaccine delivery and provides all the prerequisites for a successful needle-free vaccine delivery and also for better vaccine efficacy. Nasal cavities are also the common sites for entrance of pathogens. Furthermore, nasal administration of vaccines can lead to local production of antigen-specific sIgA, which are able to prevent pathogens from colonizing at mucosal sites (Amorij et al., 2012). Protection of antigen from enzymatic digestion, the improvement of antigen uptake by relevant cells and immune stimulation are regarded as the other advantages of nasal vaccine delivery (Pirouzmand et al., 2017). However, increasing the mucosal immunogenicity of mucosal vaccines without compromising safety and tolerability is the holy grail of vaccine industry (Sergio et al., 2014). Furthermore, antigen digestion at mucosal sites is a factor that limits the successful vaccine development. Thus, the recent studies aimed to microencapsulate different antigens within natural polymers as the carrier for the delivery of mucosal vaccines (Sergio et al., 2014; Thwala et al., 2017). Therefore, to obtain a successful mucosal immunization, efficient adjuvant, and delivery systems are required for the protection of antigens from digestion by mucosal enzymes, enhancement of antigen uptake by nasal associated lymphoid tissue (NALT) microfold cells and also improvement of the interaction between antigen and immune cells.

For mucosal administration of antigens, mucoadhesive polymers, such as chitosan, can be used as adjuvant and/or delivery carrier. Chitosan has well-defined properties, including higher bioavailability and biocompatibility, lower cost, and a higher ability to open intracellular tight junctions. Therefore, it may be a suitable polymer to be used as a delivery vehicle for mucosal vaccines (Van der Lubben et al., 2003). Several studies have been conducted on the application of chitosan in mucosal delivery of vaccines, including those by our group (Zhao et al., 2012, 2014) and other groups (Barhate et al., 2014; Khameneh et al., 2014; Arthanari et al., 2016). However, the solubility of natural polymer chitosan at the physiological pH is low (Wu et al., 2016). Functionalized forms of chitosan have attracted considerable interest to improve mucoadhesivity, permeability, stability, and controlled/extended antigen release profiles at mucosal sites (Islam et al., 2012; Mohajer et al., 2014). The present study aimed to demonstrate that biodegradable nanoparticles could serve not only as efficient vaccine antigen delivery systems, but also as potent adjuvants for stimulating humoral, cellular, and mucosal immune responses.

In general, both *in vitro* and *in vivo* behaviors of nanoparticles largely depend on their physicochemical properties, such as size, zeta potential, and surface characteristics etc. It has been shown that by reducing the particle size to the nano range, mucoadhesion property can be increased significantly (Khameneh et al., 2014). The present study also made several other improvements and advantages for nano vaccine immunization: the sizes of N-2-HACC-CMC/NDV NPs and N-2-HACC-CMC/IBV NPs are smaller than those of the particles used in previous studies, which were either larger than 300 nm (Meerak et al., 2013) or in the micron range (Singh et al., 2000). The average diameters of the both nanoparticles in this study were 251.8 ± 10.2 nm and 122.4 ± 4.2 nm (Supplementary Figure S1), zeta potentials were 46.6 ± 6.4 mV and 53.2 ± 6.68 mV, respectively (Supplementary Figure S1). Thus, they were ideal to entry into the cells. To obtain efficient antigen delivery from nasal route, the particle size must be smaller than 10 μm, with which, the nanoparticles can efficiently interact with the immune cells and have a great potential for mucoadhesion (Pirouzmand et al., 2017).

The antigen stability was also evaluated in the present study. The results indicated that the preparation processes not only protected NDV and IBV antigens from the mucosal surface clearance and enzymatic degradation, but also increased the antigen and mucosal surface contact time and improved the antigen uptake rate in the mucosa-associated lymphoid tissue. These results were in agreement with earlier findings (Arthanari et al., 2016). Furthermore, the low stability of antigens exposed to the harsh conditions in the gastrointestinal tract, together with the induction of mucosal tolerance, make the induction of a reliable immune response through the nasal delivery of assembled viral antigens very difficult (Arthanari et al., 2016). Thus, higher doses of antigens and/or very stable antigens are required. Because the CMC have a negative zeta potential, which is opposite to that of N-2-HACC, to circumvent above issues, we encapsulated NDV and IBV containing N-2-HACC with CMC, which improves both cell permeability and antigen stability (Jin et al., 2013; Dai et al., 2015) and N-2-HACC-CMC also showed good adsorption performance for the NDV and IBV antigens, enabling it to be efficiently encapsulated into nanoparticles. Thus, the loading capacity and encapsulation efficiency to vaccine antigens were higher. Also, the safety and storage stability of the nanoparticles were tested in this study. The survival rates of chickens administrated with N-2-HACC-CMC/NDV-IBV NPs and N-2-HACC-CMC/NDV/IBV NPs were 91.3 ± 2.4% and 90.8 ± 4.1%, which were much higher than that of chickens administrated with O-2’-HACC, and no significant changes in cell morphology were observed in comparison to that of the control cells, showing a much lower cytotoxicity of N-2-HACC-CMC nanoparticles. *In vivo* cytotoxicity analysis in chickens demonstrated that the feeding, drinking, and mental state of chickens were all normal, indicating that the N-2-HACC-CMC NPs as adjuvant and delivery carrier cause little cytotoxicity but have high safety level when being administrated *via* the intranasal route. Taken together, these observations reflected the proper physicochemical properties of lead formulations for vaccine delivery.

We evaluated the immunogenicity and protective efficacy of the both nanoparticles after nasal administration. A chitosan-based bioadhesive mucosal delivery system, as an intranasal vaccine adjuvant and delivery carrier for the combined attenuated live vaccine against ND and IB. Vaccination with N-2-HACC-CMC/NDV-IBV NPs and N-2-HACC-CMC/NDV/IBV NPs induced higher IgG titers against the vaccine antigen in 100% of animals (Figure 1), which completely protected the chickens from the highly virulent challenge (Supplementary Table S1). The sIgA titers in mucosa extracts were determined (Figure 2). Among the nasally immunized groups, immunization with the both nanoparticles was significantly higher than other groups (*p* < .05). It shows the potential of N-2-HACC-CMC nanoparticles for the induction of mucosal IgA against their encapsulated antigen. This could be attributed to mucoadhesion potential of N-2-HACC-CMC nanoparticles and their more prolonged presence in contact with mucosal surface (Gavini et al., 2006; Khameneh et al., 2014), which improved the antigen uptake rate by the mucosa-associated lymphoid tissue, resulting in a good mucosal immune effect. Further, the enhanced proliferation of lymphocytes has been seen in the both nanoparticle groups (Figure 3), the enhanced cellular response was also documented in the detection of the increased levels of IFN-γ and IL-2 (Figure 4), indicating a higher induction of Th-1 type responses (Jankovic & Feng, 2015).

To the best of our knowledge, this is the first study to apply chitosan derivatives in exploring the humoral, cellular, and mucosal immune response induced by intranasal administration of NDV and IBV antigens encapsulated with chitosan derivative nanoparticles. The functionalized form of chitosan used for preparing nanoparticles has attracted considerable interest due to the improved mucoadhesivity, permeability, stability, and a controlled/extended antigen release at mucosal sites (Islam et al., 2012; Sergio et al., 2014). The N-2-HACC-CMC nanoparticles show great promise and application potential as an efficient adjuvant and delivery carrier for vaccine mucosal immunity. Therefore, our future studies will explore the use of these functionalized forms of chitosan as adjuvant and delivery carrier for the preparation of mucosal vaccines and drugs.

## Conclusions

In this study, we used *N*-2-hydroxypropyl trimethyl ammonium chloride chitosan (N-2-HACC) and *N, O*-carboxymethyl chitosan (CMC) as adjuvants and delivery carriers for vaccine antigens. NDV/La Sota and IBV/H120 were encapsulated in N-2-HACC-CMC to make N-2-HACC-CMC/NDV/IBV NPs; N-2-HACC-CMC/NDV NPs and N-2-HACC-CMC/IBV NPs were mixed in a ratio of 1:1 to form N-2-HACC-CMC/NDV-IBV NPs. Both nanoparticles exhibited lower cytotoxicity and higher stability; Furthermore, their bioactivities could be maintained at 37 °C for 3 weeks and could be sustainably released from the nanoparticles after an initial burst release. Intranasal immunization of chickens with N-2-HACC-CMC/NDV/IBV NPs or N-2-HACC-CMC/NDV-IBV NPs induced higher titers of IgG and IgA antibodies, significantly promoted proliferation of lymphocytes and induced higher levels of cytokines, including IL-2, IL-4, and IFN-γ, than the commercially combined attenuated live vaccine did. This is the first study in the field of animal combined attenuated live vaccines demonstrating that intranasal administration of chickens with antigens encapsulated with chitosan derivative nanoparticles can significantly induce humoral, cellular, and mucosal immune responses. This study indicated the N-2-HACC-CMC nanoparticles show great promise and can serve as an efficient adjuvant and delivery carrier for further development of mucosal vaccines and drugs.

## Supplementary Material

IDRD_Zhao_et_al_Supplemental_Content.doc
