# Supplementary material for: Quaternized chitosan nanoparticles loaded with the combined attenuated live vaccine against Newcastle disease and infectious bronchitis elicit immune response in chicken after intranasal administration
Source: Drug Deliv. 2017 Oct 13;24(1):1574–86. doi: 10.1080/10717544.2017.1388450 (PMC8241129; doi:10.1080/10717544.2017.1388450)
Supplement: IDRD_Zhao_et_al_Supplemental_Content.doc [file IDRD_A_1388450_SM1052.doc]

**Quaternized chitosan nanoparticles loaded with the combined attenuated live vaccine against Newcastle disease and Infectious bronchitis elicit immune response in chicken after intranasally administration**

*Kai Zhao ^1, 2, *^, Shanshan Li ^1, 3^, Wei Li ^1^, Lu Yu ^1, 3^, Xutong Duan ^1, 3^, Jinyu Han ^4^, Xiaohua Wang ^1^, Zheng Jin ^4^*

*^1^ Key Laboratory of Microbiology, School of Life Science, Heilongjiang University, Harbin 150080, People’s Republic of China*

*^2^ School of Biological Science and Technology, University of Jinan, Jinan 250022, People’s Republic of China*

*^3^ Department of Avian Infectious Disease, Shanghai Veterinary Research Institute, Chinese Academy of Agricultural Sciences, Shanghai 200241, People’s Republic of China*

*^4^ Key Laboratory of Chemical Engineering Process and Technology for High-efficiency Conversion, College of Chemistry and Material Sciences, Heilongjiang University, Harbin 150080, People’s Republic of China*

**Running title:** N-2-HACC/CMC as adjuvant and delivery carrier for NDV-IBV

*Corresponding author:*

** Correspondence: Kai Zhao, Tel: +86 451 86608586, Fax: +86 451 86609016, E-mail: zybin395@126.com*

**Supplementary Information**

**Table S1** Protective efficacy of the immunized SPF chickens after being challenged with the highly virulent NDV strain F48E9 and strain IBV M41.

| **Groups** | **Strain NDV F48E9** | | | **Strain IBV M41** | | |
| --- | --- | --- | --- | --- | --- | --- |
|  | **Mortality/Total** | **Morbidity** | **Protection efficiency** | **Mortality/Total** | **Morbidity** | **Protection efficiency** |
| PBS | 7/7 | 100 % | 0 % | 7/7 | 100 % | 0 % |
| N-2-HACC-CMC NPs | 7/7 | 100 % | 0 % | 6/7 | 86 % | 0 % |
| Commercially combined attenuated live vaccine | 1/10 | 10 % | 90 % | 1/10 | 10 % | 100 % |
| N-2-HACC-CMC/NDV/IBV NPs | 0/10 | 0 % | 100 % | 1/10 | 10 % | 100 % |
| N-2-HACC-CMC/NDV-IBV NPs | 0/10 | 0 % | 100 % | 0/10 | 0 % | 100 % |

**
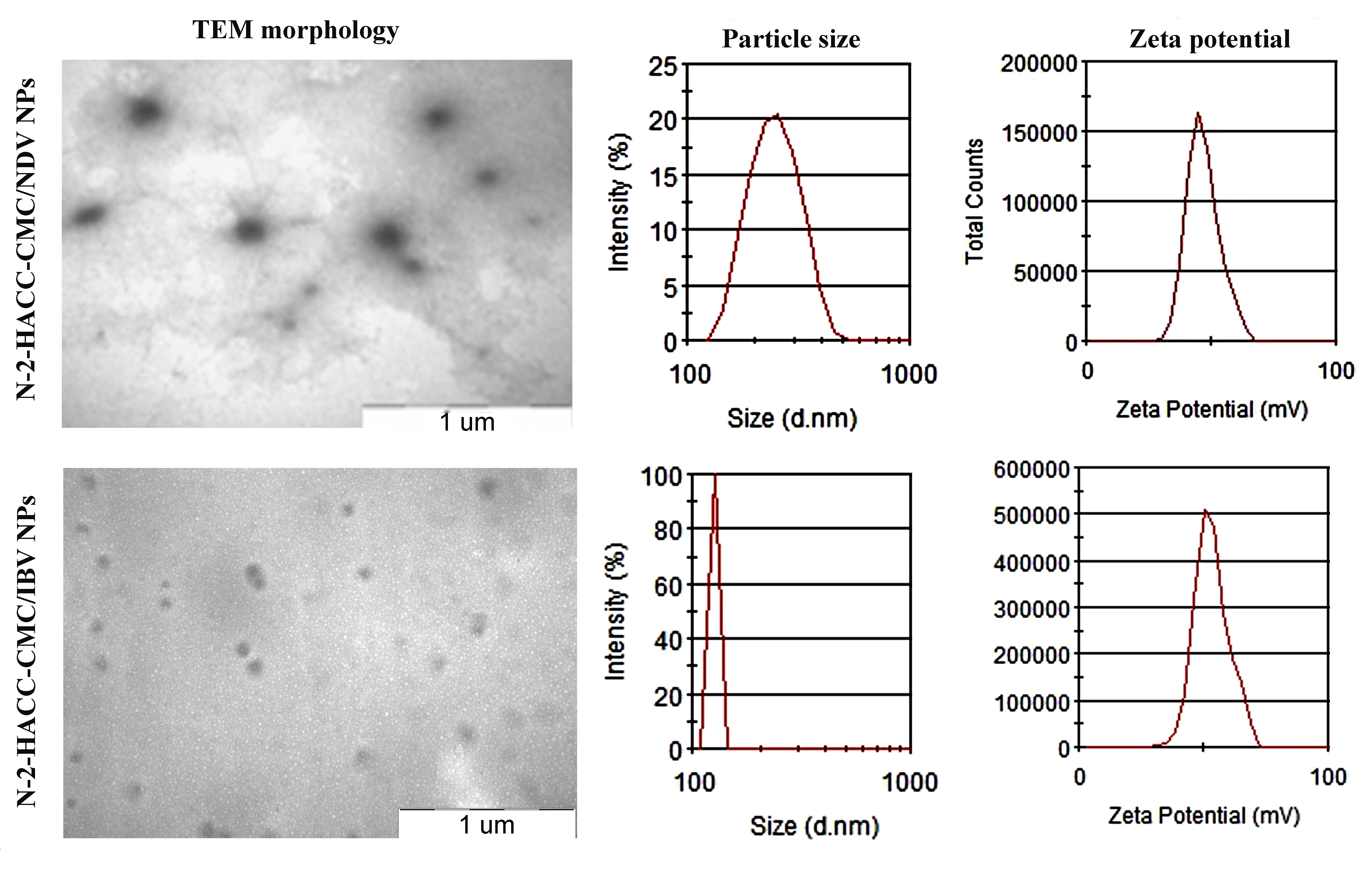
**

**Supplementary Figure S1.** The TEM morphology, particle size distribution and zeta potential of the N-2-HACC-CMC/NDV NPs and N-2-HACC-CMC/IBV NPs.

**

**

**Supplementary Figure 2.** Histopathological analyses of intestine and glandular stomach from chickens challenged with the highly virulent NDV strain F48E9 and IBV strain M41. Tissues of the intestine and glandular stomach from the SPF chickens nasally immunized with the N-2-HACC-CMC NPs and PBS.
